# Molecular Identification of Nocardia Isolates from Clinical Samples and an Overview of Human Nocardiosis in Brazil

**DOI:** 10.1371/journal.pntd.0002573

**Published:** 2013-12-05

**Authors:** Paulo Victor Pereira Baio, Juliana Nunes Ramos, Louisy Sanches dos Santos, Morgana Fonseca Soriano, Elisa Martins Ladeira, Mônica Cristina Souza, Thereza Cristina Ferreira Camello, Marcio Garcia Ribeiro, Raphael Hirata Junior, Verônica Viana Vieira, Ana Luíza Mattos-Guaraldi

**Affiliations:** 1 Universidade do Estado do Rio de Janeiro – UERJ, Faculdade de Ciências Médicas, Departamento de Microbiologia, Imunologia e Patologia, Laboratório de Difteria e Corinebactérias de Importância Clínica-LDCIC, Centro Colaborador para Difteria da CGLAB/SVS/MS, Rio de Janeiro, Rio de Janeiro, Brazil; 2 Fundação Oswaldo Cruz, Instituto Nacional de Controle de Qualidade em Saúde (INCQS), Instituto Oswaldo Cruz, Rio de Janeiro, Rio de Janeiro, Brazil; 3 Ministério da Defesa, Laboratório Químico Farmacêutico do Exército, Rio de Janeiro, Rio de Janeiro, Brazil; 4 Universidade do Estado do Rio de Janeiro – UERJ, Hospital Universitário Pedro Ernesto, Laboratório de Bacteriologia, Rio de Janeiro, Rio de Janeiro, Brazil; 5 Universidade Estadual Paulista – UNESP, Faculdade de Medicina Veterinária e Zootecnia, Botucatu, São Paulo, Brazil; University of Tennessee, United States of America

## Abstract

**Background:**

*Nocardia* sp. causes a variety of clinical presentations. The incidence of nocardiosis varies geographically according to several factors, such as the prevalence of HIV infections, transplants, neoplastic and rheumatic diseases, as well as climate, socio-economic conditions and laboratory procedures for *Nocardia* detection and identification. In Brazil the paucity of clinical reports of *Nocardia* infections suggests that this genus may be underestimated as a cause of human diseases and/or either neglected or misidentified in laboratory specimens. Accurate identification of *Nocardia* species has become increasingly important for clinical and epidemiological investigations. In this study, seven clinical *Nocardia* isolates were identified by multilocus sequence analysis (MLSA) and their antimicrobial susceptibility was also determined. Most *Nocardia* isolates were associated to pulmonary disease.

**Methodology/Principal Findings:**

The majority of Brazilian human isolates in cases reported in literature were identified as *Nocardia* sp. Molecular characterization was used for species identification of *Nocardia nova*, *Nocardia cyriacigeorgica*, *Nocardia asiatica* and *Nocardia exalbida/gamkensis*. Data indicated that molecular analysis provided a different *Nocardia* speciation than the initial biochemical identification for most Brazilian isolates. All *Nocardia* isolates showed susceptibility to trimethoprim-sulfamethoxazole, the antimicrobial of choice in the treatment nocardiosis. *N. nova* isolated from different clinical specimens from one patient showed identical antimicrobial susceptibility patterns and two distinct clones.

**Conclusions/Significance:**

Although Brazil is the world's fifth-largest country in terms of land mass and population, pulmonary, extrapulmonary and systemic forms of nocardiosis were reported in only 6 of the 26 Brazilian states from 1970 to 2013. A least 33.8% of these 46 cases of nocardiosis proved fatal. Interestingly, coinfection by two clones may occur in patients presenting nocardiosis. *Nocardia* infection may be more common throughout the Brazilian territory and in other developing tropical countries than is currently recognized and MLSA should be used more extensively as an effective method for *Nocardia* identification.

## Author Summary

Nocardiosis is an in common and potentially life-threatening infection. Most cases occur in immunocompromised patients, and a delay in establishing the diagnosis is due to the difficulties in clinical diagnosis and in cultivating and detecting *Nocardia*. Growth of *Nocardia* species in culture media is slow and incubation should be carried out for at least two weeks to detection this microorganism from clinical specimens. Accordingly, high levels of suspicion on the part of the clinician and of experience on the part of laboratory personnel are essential for detection of nocardiosis. In Brazil, clinical reports of *Nocardia* infections are scarce and the knowledge of the clinical impact of nocardiosis is fragmentary, suggesting that this genus may be underestimated as a cause of human diseases and/or neglected. In this study conventional biochemical method led to the misidentification of Brazilian isolates. Herein, molecular characterization of four loci was precisely identified as *N. nova*, *N. cyriacigeorgica*, *N. asiatica* and *N. exalbida or N. gamkensis* species. To the best of our knowledge, these are the first reported cases of human infection due to these *Nocardia* species in Brazil. Molecular methods offer a time-saving and accurate identification of the *Nocardia* genus at the species level and thus, play an important role in the diagnosis and treatment of nocardiosis.

## Introduction

Members of the *Nocardia* genus are ubiquitous environmental bacteria that can cause opportunistic infections in human and other animals [1], [2], [3]. To date, the *Nocardia* genus comprises more than 90 validly described species, including at least 30 species recognized as human opportunistic pathogens. New *Nocardia* species continue being described [2], [4].

Human nocardiosis is primarily recognized as an opportunistic disease that is intimately related to immune dysfunctions [5]. The incidence of nocardiosis varies geographically according to several factors, such as the prevalence of HIV infections, transplants, cancer and rheumatic diseases, as well as climate, socio-economic conditions and laboratory procedures for *Nocardia* detection and identification. Some reports have described an increase in the occurrence of such infections [6], [7], while others have shown that the number of nocardiosis cases has remained constant [8], [9].

Although nocardiosis typically occurs in immunosuppressed patients, infection may develop in immunocompetent patients as well. The most common clinical presentations in immunocompetent patients are superficial cutaneous disease, lymphocutaneous disease as well as mycetomas and eye infections that may occur after traumatic inoculation and are mainly described in tropical regions [6], [2].

Accurate identification of *Nocardia* species has become increasingly important for studies of antimicrobial susceptibility, clinical and epidemiological investigations. The molecular methodologies have provided a number of taxonomic changes in the *Nocardia* genus. Wallace and colleagues [10] reported that *N. asteroides* exhibited different antimicrobial susceptibility patterns. This group of bacteria known as complex *N. asteroides* is responsible for most *Nocardia* infections in humans [11]. *N. asteroides* complex was then separated and rearranged in different species: *N. asteroides*, *N. abscessus*, *N. brevicatena paucivorans* complex, *N. nova* complex (which includes *N. nova*, *N. veterana*, *N. africana*, *N. kruczakiae*), *N. transvalensis* complex, *N. farcinica* and *N*. *cyriacigeorgica*. The type VI drug pattern of *N. asteroides*, which had long been recognized as a common and significant pathogen in the United States, belonged to the *N. cyriacigeorgica* species [12]. Studies based on molecular methodologies have shown that *N. cyriacigeorgica* has been the most commonly found cause of nocardiosis in humans and animals in various parts of the world [13], [14], [15], [16], [3]. Nevertheless, other species, such as *N. farcinica*, *N. brasiliensis*, *N transvalensis*, *N. otitidiscaviarium* have also been reported frequently in nocardiosis [14], [17], [2].

The paucity of clinical reports of nocardiosis in Brazil suggests that this genus may be underdiagnosed and underestimated as a cause of human infections. Such information has led us to identify *Nocardia* species from human infection by MLSA of 16S rRNA, *gyr*B (gyrase B of the β subunit of DNA topoisomerase), *secA*1 (subunit A of SecA preprotein translocase) and *hsp*65 (65-kDa heat shock protein) genes well as to characterize their phenotypic and antimicrobial susceptibility profiles. An overview of the Brazilian reports on *Nocardia* species related to human infections was also carried out.

## Materials and Methods

### Bacterial isolation, phenotypic identification and antimicrobial susceptibility assays

Suspected *Nocardia* isolates (*n* = 7) recovered from representative clinical sites with signs and symptoms of bacterial infection were sent to a Brazilian reference laboratory (LDCIC/FCM/UERJ) over a 3 years period (from December 2007 through January 2010) for laboratory testing. Stock cultures in 10% skim milk with 25% added glycerol were maintained at −70°C and recovered as required for cultivation. The BRRJ 1046, BRRJ 1047 and BRRJ 1048 isolates were recovered from three different clinical specimens (bronchoalveolar lavage fluid - BAL, nodule secretion and tracheal aspirate, respectively) from only one patient (**Table 1**). Only clinical isolates grown in any quantity from normally sterile body fluid and/or grown in pure culture or recovered predominantly from other clinical sites were included in this study.

**Table 1:** Antimicrobial susceptibility profiles, clinical sites and phenotypic of seven *Nocardia* isolates from humans, Brazil.

		Antimicrobial profiles^a^									Phenotypic profiles^b^								
Isolate	Clinical site	AMK	GEN	TOB	ERY	CIP	AMP	AMX	IMP	TMP+SMX	45°	URE	PYR	GEL	RAM	SOR	CAS	NIT	Phenotypic Identification
1046BRRJ *	BAL^c^	S^d^	S	R^e^	S	R	#^f^	#	S	S	−	+	+	−	+	+	+	−	*N. nova*
1047BRRJ *	Nodule secretion	S	S	R	S	R	#	#	S	S	+	+	−	−	+	+	−	−	*N. asteroides*
1048BRRJ *	Tracheal aspirate	S	S	R	S	R	#	#	S	S	+	+	−	−	+	+	−	−	*N. asteroides*
1261BRRJ	Pulmonary fragment	S	S	S	R	R	#	#	S	S	+	−	−	−	−	+	−	+	*N. cyriacigeorgica*
1694BRRJ	Cerebral abscess	S	S	S	R	R	S	#	S	S	−	+	+	−	+	−	+	+	*Nocardia sp.*
2042BRRJ	BAL	S	S	S	S	S	R	R	S	S	+	−	−	−	−	+	−	+	*Nocardia sp.*
78408BRRJ	#	S	S	S	R	R	#	#	S	S	−	+	−	+	+	+	+	+	*N. pseudobrasiliensis*

a AMK, amikacin; AMX, amoxicillin; AMP, ampicillin; CIP, ciprofloxacin; ERY, erythromycin; GEN, gentamicin; IMP, imipenem; TOB, tobramycin; TMP+SMX, trimethoprim+sulfamethoxazole.

b 45°C, growth at 45°C; URE, urease production; PYR, pyrolidonyl arylamidase production; GEL, hydrolysis of gelatin; RAM, acid production on rhamnose; SOR, acid production on sorbitol; CAS, hydrolysis of casein and NIT, nitrate reduction.

c BAL - bronchoalveolar lavage fluid;

d S – sensitive;

e R – resistant;

f # Unknown;

* Clinical isolates obtained from only one patient.

The colonies grown on defibrinated sheep blood agar (5%) suggestive of the genus *Nocardia* were submitted to microscopic examination (Gram and Kinyoun acid-fast staining methods). Gram-positive branched bacilli (presenting aerial hyphae and partially acid fast bacilli) were evaluated for their ability to growth in lysozyme broth, growth at 45°C, catalase, urease and pyrolidonyl arylamidase (PYR) production, in addition to hydrolysis of casein, tyrosine, xanthine, gelatin, esculin, and hypoxanthine; acid production on glucose, adonitol, arabinose, cellobiose, dulcitol, erythritol, galactose, glycerol, inositol, lactose, maltose, mannitol, melibiose, raffinose, rhamnose, sorbitol, sucrose, trehalose, and xylose; citrate utilization and nitrate reduction [18], [19], [20], [21], [22], [12].

Susceptibility studies were performed by the diffusion disk method using ampicillin, gentamicin, tobramycin, amikacin, imipenem, ciprofloxacin, trimethoprim-sulphametaxazole (TMP-SMX) and erythromycin, in accordance with guidelines of the Clinical and Laboratory Standards Institute [18], [23].

### Molecular analysis

Molecular characterization of *Nocardia* isolates was accomplished by sequencing of the 16S rRNA, *sec*A1, *gyr*B, and *hsp*65 genes. DNA extraction, primer design, *Nocardia* gene amplification by PCR, and sequencing of amplified PCR products were performed as previously described [24], [25], [26], [27]. Sequencing reactions were performed with BigDye Terminator v 3.1 cycle sequencing kit (Applied Biosystems) on an ABI-3730 Automated DNA Sequencer (Applied Biosystems) by standard protocols. The 16S rRNA gene sequences were compared to those available in the National Center for Biotechnology Information Database (http://www.ncbi.nlm.nih.gov) using the BLAST algorithm and the Ribosomal Database Project (RDP-II) (http://rdp.cme.msu.edu). The *sec*A1, *gyr*B, *and hsp*65 gene sequences were only compared to the GenBank database.

### MLSA

The 16S rRNA, *gyr*B, *hsp*65, and *sec*A1 gene sequences were aligned by CLUSTALX [28]. The phylogenetic trees were constructed by using neighbor-joining genetic distance method using the MEGA 4.0 package with the option of complete deletion of gaps [29]. The Kimura two-parameter model was chosen for all NJ tree constructions. The reliability of each tree topology was checked by 1000 bootstrap replications.

In the BioEdit software, sequences were aligned and trimmed to define start and end positions to produce fragments of the following sizes: 1389 bp for 16S rRNA, 965 bp for *gyr*B, 401 bp for *hsp*65 and 431 bp for *sec*A1. The trimmed sequences were concatenated in the order 16S-*gyr*B-*hsp*65-*sec*A1 to generate a 3.189-bp sequence. Rooted trees obtained using individual gene sequences and concatenated sequences were generated by the neighbor-joining (NJ) algorithm with Kimura 2-parameter (K2P) correction and the maximum-parsimony (MP) algorithm in BioNumerics software. Bootstrap analysis (1000 replicates) was used to assess the robustness of the clusters.

### Pulsed-field gel electrophoresis (PFGE)

For PFGE analysis, genomic DNA was prepared using methods described by Blumel and co-workers [30] with modifications. Bacterial growth from a blood agar plate was resuspended into 1.5 ml of EC buffer (6 mM Tris-HCl [pH 7.6], 0.1 M EDTA [pH 7.6], 1 M NaCl, 1% sodium lauryl sarcosine, 0.2% sodium-deoxycholate) to a density of no. 5 McFarland standard. The purified DNA was cleaved with *Xba* I (Invitrogen) according to the manufacturer's instructions. PFGE was carried out in 0.5X TRIS-borate-EDTA-1.1% agarose gels at 13°C by a CHEF DRII system (Bio-Rad). The pulse times were 1 s to 30 s over 20 h. A lambda DNA concatemers (New England BioLabs) was used as a molecular size marker. Similarities among macrorestriction patterns were identified according to established criteria by Tenover and co-workers [31], PFGE profiles were defined as those isolates with patterns differing by more than 3 fragments. The BioNumerics Fingerprinting software (Version 4.0, Applied Math, Austin, TX) was used to confirm the findings provided by visual observation. The similarity index of the strains was calculated using the Dice correlation coefficient option of the software with a position tolerance of 1%. The unweighted-pair group method using average linkages (UPGMA) was used to construct a dendrogram.

### Nucleotide sequence accession numbers

*Nocardia* nucleotide sequences determined in this study are available under EMBL/GenBank accession numbers JQ638645 to JQ638651 for 16S rRNA gene, JQ773449 to JQ773455 for *secA*1 gene, JQ765847 to JQ765853 for *gyr*B gene and JQ782420 to JQ782426 for *hsp*65 gene.

## Results

### Phenotypic profiles

Preliminary analysis showed that microorganisms were aerobic, Gram-positive branched and filamentous bacilli and weakly acid fast by modified Kinyoun. All clinical Nocardia isolates were positive for growth in lysozyme broth catalase production and hydrolysis of esculin. The fermentation of adonitol, arabinose, cellobiose, dulcitol, erythritol, galactose, glycerol, inositol, lactose, maltose, mannitol, melibiose, raffinose, sucrose, trehalose, and xylose as well as citrate reduction, and hydrolysis of tyrosine, xanthine and hypoxanthine were negative for all clinical isolates. **Table 1** provided further biochemical results presented by the seven suspected *Nocardia* isolates recovered from representative clinical sites of patients with signs and symptoms of nocardiosis. For phenotypic identification purpose several schemes were analyzed [18], [19], [20], [21], [22], [12]. *Nocardia* species were indicated when there was an agreement among the majority of identification systems (Table 1). When not found a common outcome using different identification schemes the isolate was identified as *Nocardia* sp.

### Antimicrobial susceptibility profiles

*Nocardia* isolates showed susceptibility to amikacin, gentamicin, trimethoprim-sulphametaxazole (TMP-SMX) and imipenem; variable results were demonstrated for other antimicrobial agents tested (**Table 1**). Resistance to tobramycin was only observed for the clinical isolates identified as *N. nova* (BRRJ 1046, BRRJ 1047, BRRJ 1048) while susceptibility to erythromycin was observed for the *N. exalbida/gamkensis* BRRJ 2042 isolate.

### MLSA

**Figure 1:**
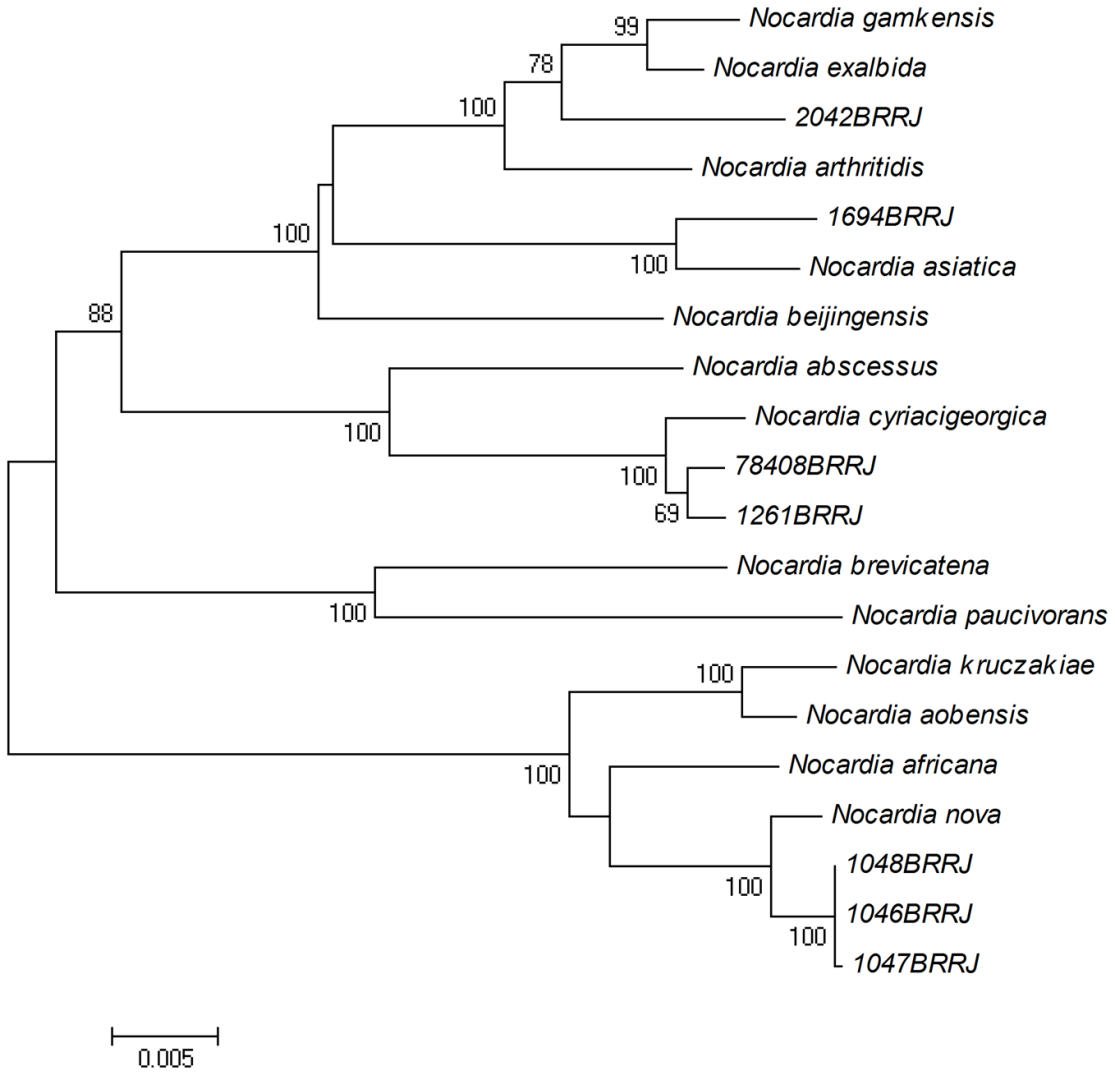
NJ tree constructed from 3,189-bp concatenated *gyrB*-16S-*secA1*-*hsp65* sequences from seven clinical isolates of *Nocardia* and those of the most closely related type species. Distance estimations were calculated by Kimura two-parameter.

**Table 2** shows the high similarity values found for the *gyr*B, 16S rRNA, *sec*A1 and *hsp*65 gene sequences of all isolates analyzed with type strains sequences. Molecular characterization by four loci (*gyr*B-16S-*sec*A1-*hsp*65) provided species identification of *N. nova* (BRRJ 1046, BRRJ 1047, BRRJ 1048 isolates), *N. cyriacigeorgica* (BRRJ 1261 and BRRJ 78408), *N. asiatica* (BRRJ 1694) and *N. exalbida/gamkensis* (BRRJ 2042). Nearly all branches of the NJ tree based on the concatenated gyrB-16S-secA1-hsp65 nucleotide sequences were supported by a bootstrap value of 100% confirming the identification of analyzed isolates (**Figure 1**). The BRRJ 2042 strain presented higher 16S rRNA sequence similarity with four *Nocardia* species: *N. exalbida*, *N. gamkensis*, *N. arthritidis* with values ranging from 99.00 to 99.51% (**Table 2**). In the phylogenetic analysis based on the concatenated sequences, the BRRJ 2042 isolate, *N. exalbida*, *N. gankensis* and *N. arthritidis* type strains appear on a branch with a high bootstrap value (100%). Even though the identification of the BRRJ 2042 isolate has not been concluded, it seems more related to *N. exalbida* and *N. gankensis*, as shown in **Figure 1**.

**Table 2:** Similarity values of the 16S rRNA, *secA1*, *hsp65* and *gyr*B gene sequences of Brazilian *Nocardia* isolates compared with *Nocardia* type strains and identification by conventional biochemical tests and using multilocus sequence analysis (MLSA).

Strain	16S rRNA similarity (%)/Type strain^1^	bp	*secA*1 similarity (%)/Type strain^2^	bp	*hsp*65 similarity (%)/Type strain^3^	bp	*gyrB* similarity (%)/Type strain^4^	bp	Phenotypic identification	MLSA identification
**1046BRRJ**	99.52 *N. nova*	1487	*99.36 N. nova*	478	99.76 *N. nova*	418	*99.14 N. nova*	1050	*N. nova*	*N. nova*
	99.07 *N. jiangxiensis*		98.72 *N. elegans*				97.90 *N. elegans*			
							89.59 *N. jiangxiensis*			
**1047BRRJ**	99.52 *N. nova*	1487	*99.36 N. nova*	497	*99.76 N. nova*	420	*98.80 N. nova*	1001	*N. asteroides*	*N. nova*
	99.08 *N. jiangxiensis*		98.72 *N. elegans*				97.50 *N. elegans*			
							89.39 *N. jiangxiensis*			
**1048BRRJ**	99.53 *N. nova*	1500	*99.36 N. nova*	491	99.75 *N. nova*	418	*99.10 N. nova*	1004	*N. asteroides*	*N. nova*
	99.07 *N. jiangxiensis*		98.72 *N. elegans*				97.80 *N. elegans*			
							89.72 *N. jiangxiensis*			
**1261BRRJ**	100,0 *N. cyriacigeorgica*	1489	100,0 *N. cyriacigeorgica*	510	100,0 *N. cyriacigeorgica*	413	100,0 *N. cyriacigeorgica*	1052	*N. cyriacigeorgica*	*N. cyriacigeorgica*
	98.90 *N. abscessus*		93.33 *N. abscessus*		97.51 *N. abscessus*		97.51 *N. abscessus*			
**1694BRRJ**	99.85 *N. asiatica*	1487	99.78 *N. asiatica*	500	100.00 *N. asiatica*	415	95.60 *N. asiatica*	1053	*Nocardia* sp.	*N. asiatica*
	99.04 *N. abscessus*		99.36 *N. abscessus*		98.78 *N. abscessus*		95.02 *N. arthritidis*			
	98.99 *N. arthritidis*		95.11 *N. arthritidis*		96.76 *N. arthritidis*		92.74 *N. abscessus*			
**2042BRRJ**	99.51 *N. exalbida*	1499	99.36 *N. arthritidis*	493	98.75 *N. gankensis*	420	96.38 *N. exalbida*	1000	*Nocardia* sp.	*N. exalbida/gankensis*
	99.23 *N. bankensis*		99.33 *N. gankensis*		98.50 *N. arthritidis*		96.17 *N. gankensis*			
	99.00 *N. arthritidis*		99.15 *N.exalbida*		98.25 *N. exalbida*		94.63 *N. arthritidis*			
**78408BRRJ**	99.93 *N. cyriacigeorgica*	1500	98.93 *N. cyriacigeorgica*	495	99.52 *N. cyriacigeorgica*	419	98.09 *N. cyriacigeorgica*	1102	*N. pseudobrasiliensis*	*N. cyriacigeorgica*
	98.90 *N. abscessus*		93.55 *N. abscessus*		97.01 *N. abscessus*		96.28 *N. abscessus*			

1 Accession numbers of 16S rRNA gene of *Nocardia* type strains: *N. abscessus*/AF218292; *N. arthritidis*/AB108781; *N. asiatica* AB092566; *N. cyriacigeorgica*/AF430027; *N. exalbida*/AB187522; *N. gankensis*/DQ235272; *N. jiangxiensis* AY639902; *N. nova*/AF430028.

2 Accession numbers of *secA*1 gene of *Nocardia* type strains: *N. abscessus*/DQ360260; *N. arthritidis*/DQ360262; *N. asiatica* DQ360263; *N. cyriacigeorgica*/DQ360272; *N. exalbida*/GU584191; *N. elegans*/DQ360273; *N. gankensis*/JN041953; *N. nova*/GU179111.

3 Accession numbers of *hsp*65 gene of *Nocardia* type strains: *N. abscessus*/DQ351152; *N. arthritidis*/JN040709; *N. asiatica* AY903631; *N. cyriacigeorgica*/HQ202353; *N. exalbida*/JN041715; *N. gankensis*/JN041716;; *N. nova*/AY756527.

4 Accession numbers of *gyr*B gene of *Nocardia* type strains: *N. abscessus*/GQ496132; *N. arthritidis*/AB450769; *N. asiatica* GU952250; *N. cyriacigeorgica*/GQ496121; *N. elegans*/GQ496116; *N. exalbida*/AB447397; *N. gankensis*/HM856182; *N. jiangxiensis* AB450792; *N. nova*/GQ496102.

### PFGE profiles

**Figure 2:**
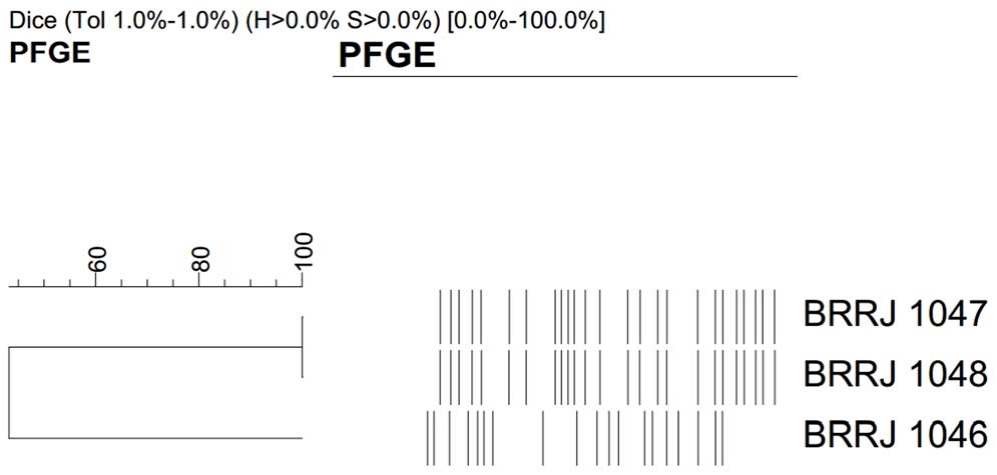
Dendogram displaying PFGE profiles of four *Nocardia nova* isolates identified in this study.

The PFGE analysis was performed to determine the genetic relatedness of *N. nova* isolates. The restriction endonuclease *Xba*I revealed two distinct PFGE profiles among the *N. nova* isolates, which were designated A (BRRJ 1046/BAL isolate) and B (BRRJ 1047 and BRRJ 1048/nodule secretion and tracheal aspirate isolates, respectively) (**Figure 2**).

### Review of the Brazilian literature

In the present study, a literature search was performed in PubMed and SciElo Brasil databases using the key words ‘Nocardia’ or ‘Nocardiosis’ and ‘Brazil’ and encompassing articles published from 1970 to March 1, 2013. The review of the literature in both English and Portuguese yielded a summary of some microbiological and clinical aspects of clinical cases of Nocardia, excluding mycetomas cases as presented in **Table 3**. A total of 27 studies concerning 58 cases of *Nocardia* infection were found available in the literature during the 43 years period.

**Table 3:** Characteristics of 27 previous studies of nocardiosis in Brazil from 1970 to 2013.

City/State (Year)^References^	Nocardiosis disease (number of cases)	Underlying conditions or associated conditions of immunosuppression (number of cases)	Identification	Therapy^a^	Outcome
Porto Alegre/RS (1978) ^[83]^	Pulmonary (2)	Tobacco smoking (1); Neoplasic disease, corticotherapy (1)	*N. asteroides; N. asteroids*	SUF, CS; SUF	Cure (1); Death (1)
São Paulo/SP (1989) ^[84]^	Cerebrospinal fluid (1)	HIV infection	*Nocardia* sp.	-	-
Salvador/BA (1990) ^[85]^	Pulmonary (6)	Not identified	*Nocardia* sp.	-	-
Ribeirão Preto/SP [1993 (1968–1991)] ^[42]^	Pulmonary (6); Disseminated (3)	Renal transplant, corticotherapy (9)	*Nocardia* sp. (3); *N. asteroides* (5); *N. brasiliensis* (1)	TMP+SMX (associations)	Death (7); Cure (2)
Santa Maria/RS (1993) ^[86]^	Peritonitis (1)	Systemic lupus erythematous and failure renal, ambulatory peritoneal dialysis	*N. asteroids*	CET/TMP+SMX	Cure
São Paulo/SP (1995) ^[87]^	Cerebelar abscessus and pulmonary (1)	HIV infection	*N. asteroides*	CTR	Death
São Paulo/SP (1995) ^[62]^	Keratitis (1)	Myopic keratomileusis	*N. asteroides*	-	Visual debilitating sequelae
São Paulo/SP (1997) ^[88]^	Disseminated (1)	Bone marrow transplant	*Nocardia* sp.	TMP+SMX	Cure
São Paulo/SP (1997) ^[89]^	Pulmonary (1)	Alcoholism, tobacco smoking, COPD^b^	*Nocardia* sp.	TMP+SMX, CTR, CM	Death
Santa Maria/RS (1999) ^[80]^	Disseminated (1)	HIV infection	*N. pseudobrasiliensis*	AMB/CM, AMK/IMP,TMP+SMX	Death
Uberaba/MG (2000) ^[90]^	Brain abscessus and disseminated (1)	Autoimmune haemolytic anaemia and thrombocytopenic purpura (Evans Syndrome), corticotherapy	*Nocardia* sp.	CM, CFPM VAN/IMP, CIL, AMB/AZ, TMP+SMX	Cure
Niterói/RJ (2002) ^[91]^	Pulmonary (1)	HIV infection, healed tuberculosis	*Nocardia* sp.	TMP+SMX	Death
Campinas/SP (2003) ^[63]^	Scleritis (1)	None	*N. asteroides*	TMP+SMX and AMK eyedrops	Visual debilitating sequelae
São Paulo/SP (2004) ^[64]^	Keratitis (1)	Implantation of intracorneal rings segments – IRS	*Nocardia* sp.	-	Cure; No remotion of IRS
Porto Alegre/RS (2005) ^[81]^	Disseminated and thyroid abscessus (1)	Corticotherapy	*N. farcinica*	TMP+SMX	Death
São Paulo/SP (2006) ^[92]^	Pulmonary (1)^c^	HIV infection	*Nocardia* sp.	AMB/TMP+SMX	Death
São Paulo/SP (2006) ^[93]^	Pulmonary and cutaneous (1)	Bronchiolitis obliterans, corticotherapy	*N. asteroids*	TMP+SMX	Cure
Niterói/RJ (2007) ^[65]^	Scleritis (1)	Keratoplasty and intraocular lens implantation		TMP+SMX, AMK eyedrops	
Porto Alegre/RS (2007) ^[41]^	Pulmonary (14)	COPD (3), systemic lupus erythematous (1), HIV infection (1), neoplasic disease (4), corticotherapy (10), radiotherapy (4), chemotherapy (6), liver transplant (1), kidney transplant (2), asthma (1), chronic bronchitis (1)	*N. asteroides* complex (4); *N. asteroides* (1); *Nocardia* sp.(9)	TMP+SMX	Cure (6); Death (8);
	Pulmonary and cutaneous (1)	Not identified	Nocardia sp. (1)	TMP+SMX	Death (1);
	Disseminated (4)	COPD (1), diabetes (1), corticotherapy (3), neoplasic disease (1), chemotherapy (2), radiotherapy (1), liver transplant (1), not identified (1)	*Nocardia* sp. (4)	IMP+VAN/SUF+AMK/TMP+SMX	Death (2); Cure (2)
São Paulo/SP (2007) ^[94]^	Pulmonary (1)	Idiopathic thrombocytopenic purpura, corticotherapy	*N. farcinica*	TMP+SMX	Death
Niterói/RJ (2008) ^[95]^	Pulmonary (1)	COPD, bronchiectasis, corticotherapy	*Nocardia* sp.	AMK, IMP,CIL/TMP+SMX	Cure
Campo Grande/MS (2008) ^[96]^	Pulmonary (1)	Not identified	*Nocardia brasiliensis*.	-	-
Niterói/RJ (2009) ^[97]^	Pulmonar nocardiosis (1)	HIV infection	*Nocardia* spp.	Antiretroviral drugs TMP+SMX	Cure
Niterói/RJ (2009) ^[98]^	Pulmonary abscesso (1)	Chronic lymphocytic leukemia	*Nocardia brasiliensis*	TMP+SMX	Cure
São Paulo/SP (2011)^[99]^	Endocarditis (1)	Liver transplant, corticotherapy	*Nocardia* sp.	CSP, AZ/IMP, AMK, TMP+SMX	Cure
São Paulo/SP (BH/Fortaleza/Uberlândia) (2011) ^[45]^	Disseminated (1)	Kidney transplant	*Nocardia* sp.	TMP+SMX	Cure
São Paulo/SP (2012) ^[52]^	Pneumocystis, fungal infections or nocardiosis^d^ (20)	Not identified	*Nocardia* sp.	Not identified	Not identified

a AMB, amphotericin B; AMK, amikacin; AZ, azathioprine, CET, cephalothin; CFPM, cefepime; CIL, cilastatin; CM, clindamycin; CS, cycloserine; CSP, cyclosporine; CTR, ceftriaxone; IMP, imipenem; SUF, sufadiazine; TMP+SMX, trimethoprim+sulfamethoxazole; VAN, vancomycin.

b COPD Chronic obstructive pulmonary disease.

c Coinfection with *M. tuberculosis* and *Aspergillus* sp.

d Bronchoscopy diagnosis of pulmonary tuberculosis in patients with negative sputum smear microscopy results.

## Discussion

Definitive bacteriological diagnosis of nocardiosis depends upon the isolation and identification of the causal agent from clinical material as well as the laboratory in which the specimens are analyzed. Growth of *Nocardia* species in culture media is slow and incubation should be carried out for at least two weeks [5], [2]. Premature discontinuation of the culture will decrease the sensitivity of recovery and may contribute to underestimation of the true incidence of nocardiosis. Most of the laboratories discard bacterial cultures which are negative after 48 h and *Mycobacterium tuberculosis* (TB) laboratories do not process sputum specimens without decontaminating non-mycobacterial pulmonary pathogens [32], [33].

Furthermore, modified acid-fast (Kinyoun) and Gram staining of specimens are particularly important to provide a rapid, economical and presumptive diagnosis while awaiting the results of the culture [34], [5]. As opposed to mycobacteria, *Actinomyces* can be more easily differentiated from *Nocardia* as they are not stained by modified acid-fast stain [22], [6], [35], [36].

In accordance to Kiska and co-workers [18] no single method could accurately identify all *Nocardia* species associated with human and animal infections. In that opportunity, a combination of the antimicrobial susceptibility pattern, colony pigment and a group of biochemical tests was suggested to identify all isolates at the species level.

However, most recent studies revealed that *Nocardia* speciation might require confirmation by molecular techniques, which may change the initial biochemical identification [22], [6], [37]. Thus, various molecular methods have been proposed to provide accurate *Nocardia* species identification [17], [14], [25]. Sequence analysis of 16S rRNA performed by Liu and co-workers [37] showed that phenotypic identification assays produced 37% misidentifications of *Nocardia* species. Although the 16S rRNA gene sequence has been broadly used to discriminate *Nocardia* species, misidentification of microorganisms may occur due to high sequence similarity and multiple although different copies of this gene [2], [38]. In attempt to improve the identification of the increasing number of species within *Nocardia* genus, the analysis of other housekeeping genes such as the 65-kDa heat shock protein gene (*hsp65*), essential secretory protein A (*secA*1), gyrase B (*gyrB*) has also been performed [14], [25], [26], [27]. Sequence analysis of multiple housekeeping genes provided more informative nucleotide sites and buffers against the distorting effects of homologous recombination and horizontal gene transfer of a single gene [38], [39]. In this context, MLSA has been regarded as an alternative technique for the identification and classification of a diverse group of bacteria, including the *Nocardia* genus [14], [40].

As in many other developing countries, nocardiosis prevalence is still unknown in Brazil. To our knowledge, this is the first report of the identification of nocardia species by MLSA in our country. In order to collect information on this Public Health issue, a complete overview of Brazilian published case reports of nocardiosis, excluding mycetomas is presented herein (**Table 3**). The only large series of nocardial infections occurred from 1978 to 1998 and was reported by Chedid and co-workers [41]. Solid organ transplantation was the most common underlying condition before the advent of effective medical therapy, which included the introduction of cyclosporine and prophylaxis with TMP-SMZ [5], [42], [43], [44]. Batista and co-workers [45] found only one case of nocardiosis among 1046 kidney and 708 liver transplants patients registered in four Brazilian centers in different geographical areas from 2001 to 2006. In those institutions, cotrimozaxole prophylaxis was routinely used for 6 months following transplantation and in situations where there was an increase in immunosuppressive therapy for rejection.

Most of the Brazilian studies (39 cases, 67.24%) indicated pulmonary disease as the major clinical presentation of nocardiosis in our country (**Table 3**). In some developing countries, where other chronic lung diseases, particularly TB are prevalent, pulmonary nocardiosis may be more common than is currently recognized, especially in areas with HIV-associated tuberculosis. One of the reasons for this occurrence is that the pulmonary manifestation of nocardiosis is often confused with TB [33], [35]. Clinical, radiological and histopathological findings are not sufficient for the recognition of pulmonary nocardiosis, suggesting that a considerable percentage of patients presenting symptoms of chronic lung disease could be suffering from pulmonary nocardiosis [5], [46]. In some African countries, where HIV-related tuberculosis occurs frequently, there are reports of a high prevalence of nocardiosis [33], [47], [48]. Another issue for the recognition of pulmonary nocardiosis refers to the difficulty of diagnosing in the laboratory. Some authors have emphasized that in regions where HIV-related tuberculosis occurs, *Nocardia* strains are missed or misidentified in clinical specimens and it is possible that some patients diagnosed as smear-negative pulmonary TB actually have nocardiosis [33], [47], [48], [49], [50], [51].

In Brazil, Jacomelli and co-workers [52] investigated 286 patients with clinical or radiological suspicion of TB who were unable to produce sputum or had a negative smear. They found that 7% of infections were caused by *Pneumocystis*, fungi and *Nocardia*. In 2011, the incidence of tuberculosis in Brazil was 37.2/100,000 inhabitants, however, there were Brazilian cities where the incidence of tuberculosis was much higher that registered in the city of São Paulo city (39.3/100,000) including Rio de Janeiro (70.7/100,000 inhabitants), Porto Alegre (109.2/100,000), Recife (93.2/100,000) among others [53], [54]. Unfortunately, there are no other studies on microbiological aspects of the infections diagnosed as smear-negative pulmonary TB, which should be evaluated in different states of Brazil.

Brazil's AIDS treatment program has been cited widely as the developing world's largest and most successful AIDS treatment program. The program guarantees free access to highly active antiretroviral therapy (HAART) for all people living with HIV/AIDS in need of treatment [55], [56]. This may reflect in low number of reports of cases of nocardiosis in patients with HIV in Brazil.

Pulmonary and disseminated nocardiosis have also been recently reported in immunocompetent patients in different countries [33], [57], [58], [59]. Although frequent in India, cases of keratitis are relatively rare in another countries [60], [61]. In Brazil, only four cases of eye infection due to *Nocardia* have been reported [62], [63], [64], [65]. Mycetomas cases caused by *Nocardia* sp. have been described in São Paulo and other cities [41], [66], [67], [68], [69], [70], [71], [72], [73], [74], [75].

*Nocardia* species differ in their responses to antimicrobials and susceptibility tests for all clinically significant *Nocardia* isolates are recommended. However, due to the slow growth of these bacteria, clinicians usually begin treatment empirically before these results are made available [13]. Nocardiosis treatment is usually prolonged and TMP-SMX is the most widely prescribed for therapy of nocardiosis [13], [22], [76]. For patients with serious diseases clinicians recommend a three-drug regimen consisting of TMP-SMX, amikacin, and either ceftriaxone or imipenem. There has not been any report of resistance to this combination as of yet [2], [8]. In our study, 92% of isolates were sensitive to imipenem and 100% were sensitive to amikacin and TMP-SMX. Some reports have described high levels of sulfonamide resistance among numerous *Nocardia* species [77], [78]. Nevertheless, these values have been contested by Brown-Elliott and co-workers [76] that suggested that these findings may be associated with difficulty in the laboratory interpretation of *in vitro* MICs for TMP-SMX and sulfamethoxazole. Nowadays, TMP-SMX remains the drugs of choice for nocardiosis treatment and prophylaxis against *Nocardia* infection in immunocompromised patients [44], [76], [79].

The reported Brazilian cases displayed in **Table 3** showed that the majority (55.56%) of the isolates were identified as *Nocardia* sp. In the present investigation, the use of varied conventional biochemical algorithms described by different authors [18], [19], [20], [21], [22] led to the misidentification of five out of seven of the Brazilian isolates tested.

In Brazil, molecular analysis for identification of *Nocardia* species was carried out on only a few occasions [3], [80], [81]. In the present study, the identification system based on MLSA methodology was capable of differentiating currently recognized *Nocardia* species. Data indicated that all *Nocardia* isolates were identified by phylogenetic analysis based on the concatenated gyrB-16S-secA1 hsp65 gene sequences as recommended by Mc Taggart and co-workers [14]. MLSA has provided the identification of the following species: *N. nova, N. cyriacigeorgica, N. asiatica and N. exalbida/gamkensis*. Most of the species were related with pulmonary disease, except for *N. asiatica* which was isolated from a patient with a brain abscess. To our knowledge, this is the first Brazilian report of human isolates of *N. cyriacigeorgica*. Two isolates were identified by MLSA as *N. cyriacigeorgica*, including one isolated from a patient with pulmonary disease. In Brazil, *N. cyriacigeorgica* had previously only been isolated from bovine bulk tank milk [3].

Similar to observations performed by Mc Taggart and co-workers [14], MLSA did not distinguish the *N. arthritidis*, *N. gamkensis*, and *N. exalbida* type strains. Although the BRRJ 2042 strain seemed more related to *N. exalbida* and *N. gamkensis* species, they formed a cluster together with *N. arthritidis* supported by a bootstrap of 100%. While sequence analysis of additional genes may demarcate these type strains, failure to do so would prompt an extensive evaluation of the legitimacy of their species status. Mc Taggart and colleagues [14] demonstrated that the MLSA scheme revealed two sets of type strains that failed to form distinct clusters. One of these sets was comprised by *N. arthritidis* DSM 44731T, *N. gamkensis* DSM 44956T, *N. exalbida* DSM 44883T and 7 clinical isolates formed a cluster with 98% bootstrap support.

This study also made the analysis of the genetic relationship of *N. nova* isolates recovered from three different clinical specimens of a from the same patient by the PFGE method. Surprisingly, we observed that the patient presented pulmonary coinfection by two *N. nova* clones, one of which (PFGE profile B) was disseminated and also detected in the nodular discharge.

Interestingly, the overview of literature, nocardiosis was only reported cases in 6 of the 26 Brazilian states. During the last decade, only 14 cases were reported in the states of Pernambuco, Goiás, São Paulo, Rio de Janeiro and Rio Grande do Sul. Data highlight the fact that nocardiosis remains underdiagnosed in most of our country presents continental dimensions and large socioeconomic differences.

Therefore, knowledge of the clinical impact of nocardiosis remains scarce and fragmentary mainly due to the difficulties in clinical and laboratorial diagnosis. Reports have suggested that there is usually a delay in the diagnosis of nocardiosis which is attributed to difficulties to clinical, radiological and microbiological diagnose. The usual reason for requesting culture studies for the detection of *Nocardia* spp. is that a patient has not responded to the usual anti-bacterial or anti-TB treatment [13], [82]. In conformity with Wilson [11], the isolation of *Nocardia* from the respiratory tract or other body source, independent of the immunologic status of the patients, should not be discarded as a contaminant or commensal organism. In case of difficulties in the identification of *Nocardia*, the suspected isolates should be conducted to a Clinical Reference Laboratory. Optimal therapeutic strategies depend on rapid and accurate identification of *Nocardia* species. In this context, molecular methods for identification, such as MLSA analysis offers a timesaving alternative to conventional methods for identifying the *Nocardia* genus at the species level, both in Brazil and abroad.

## References

[1] Ribeiro MG, Salerno T, Mattos-Guaraldi AL, Camello TC, Langoni H, et al (2008) Nocardiosis: an overview and additional report of 28 cases in cattle and dogs. Rev Inst Med Trop Sao Paulo 50: 177–185. 18516465 10.1590/s0036-46652008005000004

[2] Conville PS, Witebsky F (2011) *Nocardia*, *Rhodococcus*, *Gordonia*, *Actinomadura*, *Streptomyces*, and Other Aerobic Actinomycetes. In: Versalovic, J, Carroll, K C., Funke, G, Jorgensen, J H., Landry, M L., Warnock, D. W., Manual of Clinical Microbiology 10^th^. Ed. ASM Press, Washington. D. C.

[3] Condas LAZ, Ribeiro MG, Gono T, Matsuzawa T, Yazawa K, et al (2012) Molecular identification and thermoresistance to boiling of *Nocardia farcinica* and *Nocardia cyriacigeorgica* from bovine bulk tank milk. Brazilian J Microbiol 43: 1038–1041. 10.1590/S1517-838220120003000029 PMC3768902 24031926

[4] Euzéby JP (2013) List of Prokaryotic names with Standing in Nomenclature. Available: http://www.bacterio.net

[5] Clark NM (2009) AST Infectious Diseases Community of Practice (2009) Nocardia in Solid Organ Transplant Recipients. Am J Transplant 9 (Suppl 4? S70–S77. 20070699 10.1111/j.1600-6143.2009.02896.x

[6] Ambrosioni J, Lew D, Garbino J (2010) Nocardiosis: updated clinical review and experience at a tertiary center. Infection 38: 89–97. 20306281 10.1007/s15010-009-9193-9

[7] Bibi S, Irfan S, Zafar A, Khan E (2012) Isolation frequency and susceptibility patterns of Nocardia species at a tertiary hospital laboratory in Karachi, Pakistan. J Infect Dev Ctries 5: 499–501. 10.3855/jidc.1278 21727653

[8] Al-Jahdali H, Baharoon S, Alothman S, Memish Z, Waness A (2011) Nocardiosis in a Tertiary Care Hospital in Saudi Arabia. J Glob Infect Dis 3: 128–132. 21731298 10.4103/0974-777X.81688 PMC3125024

[9] Hardak E, Yigla M, Berger G, Sprecher H, Oren I (2012) Clinical spectrum and outcome of Nocardia infection: experience of 15-year period from a single tertiary medical center. Am J Med Sci 343: 286–90. 21825961 10.1097/MAJ.0b013e31822cb5dc

[10] Wallace RJ Jr, Steele LC, Sumter G, Smith JM (1988) Antimicrobial susceptibility patterns of *Nocardia asteroides* . Antimicrob Agents Chemother 32: 1776–1779. 3072921 10.1128/aac.32.12.1776 PMC176016

[11] Wilson JW (2012) Nocardiosis: updates and clinical overview. Mayo Clin Proc 87: 403–7. 22469352 10.1016/j.mayocp.2011.11.016 PMC3498414

[12] Conville PS, Witebsky FG (2007) Organisms designated as *Nocardia asteroides* drug pattern type VI are members of the species *Nocardia cyriacigeorgica* . J Clin Microbiol 45: 2257–2259. 17475753 10.1128/JCM.00133-07 PMC1932980

[13] Minero MV, Marín M, Cercenado E, Rabadán PM, Bouza E, et al (2009) Nocardiosis at the turn of the century. Medicine (Baltimore) 88: 250–261. 19593231 10.1097/MD.0b013e3181afa1c8

[14] McTaggart LR, Richardson SE, Witkowska M, Zhang SX (2010) Phylogeny and identification of *Nocardia* species on the basis of multilocus sequence analysis. J Clin Microbiol 48: 4525–4533. 20844218 10.1128/JCM.00883-10 PMC3008441

[15] Kong F, Wang H, Zhang E, Sintchenko V, Xiao M, et al (2010) secA1 gene sequence polymorphisms for species identification of *Nocardia* species and recognition of intraspecies genetic diversity. J Clin Microbiol 48: 3928–3934. 20810768 10.1128/JCM.01113-10 PMC3020853

[16] Chen YC, Lee CH, Chien CC, Chao TL, Lin WC, et al (2012) Pulmonary nocardiosis in southern Taiwan. J Microbiol Immunol Infec 24 pii: S1684–1182(12)00161-2 doi:[]10.1016/j.jmii.2012.07.017. [Epub ahead of print] 10.1016/j.jmii.2012.07.017 23017691

[17] Tan CK, Lai CC, Lin SH, Liao CH, Chou CH, et al (2010) Clinical and microbiological characteristics of Nocardiosis including those caused by emerging *Nocardia* species in Taiwan, 1998–2008. Clin Microbiol Infect 16: 966–72. 19860823 10.1111/j.1469-0691.2009.02950.x

[18] Kiska DL, Hicks K, Pettit DJ (2002) Identification of medically relevant *Nocardia* species with an abbreviated battery of tests. J Clin Microbiol 40: 1346–1351. 11923355 10.1128/JCM.40.4.1346-1351.2002 PMC140358

[19] Brown JM, McNeil MM (2003) *Nocardia*, *Rhodococcus*, *Gordonia*, *Actinomadura*, *Streptomyces*, and other aerobic actinomycetes. In: P. R Murray, E. J Baron, J. H Jorgensen, M. A Pfaller, and R. H Yolken, Manual of clinical microbiology 8th ed. ASM Press, Washington, D.C. p. 370–398.

[20] Roth A, Andrees S, Kroppenstedt RM, Harmsen D, Mauch H (2003) Phylogeny of the genus *Nocardia* based on reassessed 16S rRNA gene sequences reveals underspeciation and division of strains classified as *Nocardia asteroides* into three established species and two unnamed taxons. J Clin Microbiol 41: 851–6. 12574299 10.1128/JCM.41.2.851-856.2003 PMC149683

[21] Wauters G, Avesani V, Charlier J, Janssens M, Vaneechoutte M, et al (2005) Distribution of nocardia species in clinical samples and their routine rapid identification in the laboratory. J Clin Microbiol 43: 2624–8. 15956375 10.1128/JCM.43.6.2624-2628.2005 PMC1151960

[22] Brown-Elliott BA, Brown JM, Conville PS, Wallace RJ Jr (2006) Clinical and laboratory features of the *Nocardia* spp. based on current molecular taxonomy. Clin Microbiol Rev 19: 259–282. 16614249 10.1128/CMR.19.2.259-282.2006 PMC1471991

[23] CLSI Clinical Laboratory Standards Institute (2007) Methods for antimicrobial dilution and disk susceptibility testing of infrequently isolated or fastidious bacteria. M45-A, CLSI, Wayne, 15 pp. 10.1086/510431 17173232

[24] Baio PVP, Mota HF, Freitas AD, Gomes DL, Ramos JN, et al (2013) Clonal multidrug-resistant Corynebacterium striatum within a nosocomial environment, Rio de Janeiro, Brazil. Mem Inst Oswaldo Cruz 108: 23–29. 23440110 10.1590/S0074-02762013000100004 PMC3974316

[25] Conville PS, Zelany AM, Witebsky FG (2006) Analysis of secA1 gene sequences for identification of *Nocardia* species. J Clin Microbiol 44: 2760–2766. 16891489 10.1128/JCM.00155-06 PMC1594632

[26] Yin X, Liang S, Sun X, Luo S, Wang Z, et al (2007) Ocular Nocardiosis: *hsp*65 Gene Sequencing for Species Identification of *Nocardia* spp. American J Ophthalmology 144: 570–573. 10.1016/j.ajo.2007.06.031 17698022

[27] Takeda K, Kang Y, Yazawa K, Gonoi T, Mikami Y (2010) Phylogenetic studies of *Nocardia* species based on *gyr*B gene analyses. J Med Microbiol 59: 165–171. 19833784 10.1099/jmm.0.011346-0

[28] Thompson JD, Gibson TJ, Plewniak F, Jeanmougin F, Higgins DG (1997) The ClustalX windows interface: flexible strategies for multiple sequence alignment aided by quality analysis tools. Nucleic Acids Res 25: 4876–4882. 9396791 10.1093/nar/25.24.4876 PMC147148

[29] Tamura K, Dudley J, Nei M, Kumar S (2007) MEGA4: Molecular Evolutionary Genetics Analysis (MEGA) software version 4.0. Mol Biol Evol 24: 1596–1599. 17488738 10.1093/molbev/msm092

[30] Blümel J, Blümel E, Yassin AF, Schmidt-Rotte H, Schaal KP (1998) Typing of Nocardia farcinica by pulsed-field gel electrophoresis reveals an endemic strain as source of hospital infections. J Clin Microbiol 36: 118–22. 9431933 10.1128/jcm.36.1.118-122.1998 PMC124820

[31] Tenover FC, Arbeit RD, Goering RV, Mickelsen PA, Murray BE, et al (1995) Interpreting chromosomal DNA restriction patterns produced by pulsed-field gel electrophoresis: criteria for bacterial strain typing. J Clin Microbiol 33: 2233–2239. 7494007 10.1128/jcm.33.9.2233-2239.1995 PMC228385

[32] Agterof MJ, van der Bruggen T, Tersmette M, ter Borg EJ, van den Bosch JM, et al (2007) Nocardiosis: a case series and a mini review of clinical and microbiological features. Neth J Med 65: 199–202. 17587645

[33] Alnaum HM, Elhassan MM, Mustafa FY, Hamid ME (2011) Prevalence of Nocardia species among HIV-positive patients with suspected tuberculosis. Trop Doct 41: 224–226. 21878441 10.1258/td.2011.110107

[34] Peleg AY, Husain S, Qureshi ZA, Silveira FP, Sarumi M, et al (2007) Risk factors, clinical characteristics, and outcome of Nocardia infection in organ transplant recipients: a matched case-control study. Clin Infect Dis 44: 1307–1314. 17443467 10.1086/514340

[35] Sullivan DC, Chapman SW (2010) Bacteria that masquerade as fungi: actinomycosis/nocardia. Proc Am Thorac Soc 7: 216–221. 20463251 10.1513/pats.200907-077AL

[36] Savini V, Fazii P, Favaro M, Astolfi D, Polilli E, et al (2012) Tuberculosis-like pneumonias by the aerobic actinomycetes Rhodococcus, Tsukamurella and Gordonia. Microbes Infect 14: 401–410. 22192786 10.1016/j.micinf.2011.11.014

[37] Liu WL, Lai CC, Ko WC, Chen YH, Tang HJ, et al (2011) Clinical and microbiological characteristics of infections caused by various Nocardia species in Taiwan: a multicenter study from 1998 to 2010. Eur J Clin Microbiol Infect Dis 30: 1341–1347. 21461846 10.1007/s10096-011-1227-9

[38] Conville PS, Witebsky FG (2005) Multiple copies of the 16S rRNA gene in Nocardia nova isolates and implications for sequence-based identification procedures. J Clin Microbiol 43: 2881–2885. 15956412 10.1128/JCM.43.6.2881-2885.2005 PMC1151890

[39] Gevers D, Cohan FM, Lawrence JG, Spratt BG, Coenye T, et al (2005) Opinion: Re-evaluating prokaryotic species. Nat Rev Microbiol 3: 733–739. 16138101 10.1038/nrmicro1236

[40] Tamura T, Matsuzawa T, Oji S, Ichikawa N, Hosoyama A, et al (2012) A genome sequence-based approach to taxonomy of the genus *Nocardia* . Antonie Van Leeuwenhoek 102: 481–491. 22836677 10.1007/s10482-012-9780-5

[41] Chedid MB, Chedid MF, Porto NS, Severo CB, Severo LC (2007) Nocardial infections: report of 22 cases. Rev Inst Med Trop Sao Paulo 49: 239–246. 17823754 10.1590/s0036-46652007000400009

[42] Santamaria Saber LT, Figueiredo JF, Santos SB, Levy CE, Reis MA, et al (1993) Nocardia infection in renal transplant recipient: diagnostic and therapeutic considerations. Rev Inst Med Trop Sao Paulo 35: 417–21. 8115809 10.1590/s0036-46651993000500006

[43] Kanne JP, Yandow DR, Mohammed TL, Meyer CA (2011) CT findings of pulmonary nocardiosis. AJR Am J Roentgenol 197: W266–W272. 21785052 10.2214/AJR.10.6208

[44] Yu X, Han F, Wu J, He Q, Peng W, et al (2011) *Nocardia* infection in kidney transplant recipients: case report and analysis of 66 published cases. Transpl Infect Dis 13: 385–391. 21824241 10.1111/j.1399-3062.2011.00607.x

[45] Batista MV, Pierrotti LC, Abdala E, Clemente WT, Girão ES, et al (2011) Endemic and opportunistic infections in Brazilian solid organ transplant recipients. Trop Med Int Health 16: 1134–1142. 21692958 10.1111/j.1365-3156.2011.02816.x

[46] Santos M, Gil-Brusola A, Morale P (2011) Infection by Nocardia in solid organ transplantation: thirty years of experience. Transplant Proc 43: 2141–2144. 21839216 10.1016/j.transproceed.2011.06.065

[47] Rasheed MU, Belay G (2008) Nocardiosis in HIV seropositive clinically suspected pulmonary tuberculosis patients. Trop Doct 38: 34–35. 18302863 10.1258/td.2007.060055

[48] Nwuba CO, Kogo G, Ogbu N, Abolarin O, Okonkwo R (2012) Nocardiosis - an emerging complication in the clinical management of HIV infected patients. Retrovirology (Suppl 1? P134 http://www.retrovirology.com/content/9/S1/P134.

[49] Baily GG, Neill P, Robertson VJ (1988) Nocardiosis: a neglected chronic lung disease in Africa? Thorax 43: 905–910. 3065975 10.1136/thx.43.11.905 PMC461554

[50] Poonwan N, Kusum M, Mikami Y, Yazawa K, Tanaka Y, et al (1995) Pathogenic Nocardia isolated from clinical specimens including those of AIDS patients in Thailand. Eur J Epidemiol 11: 507–512. 8549723 10.1007/BF01719301

[51] Ba-Fall KM, Mbaye MN, Niang AR, Faye ME, Fall K, et al (2011) [Nocardiosis: 4 cases in Senegal]. Med Trop (Mars) 71: 613–614. 22393631

[52] Jacomelli M, Silva PR, Rodrigues AJ, Demarzo SE, Seicento M, et al (2012) Bronchoscopy for the diagnosis of pulmonary tuberculosis in patients with negative sputum smear microscopy results. J Bras Pneumol 38: 167–173. 22576423 10.1590/s1806-37132012000200004

[53] Boletim Epidemiológico. Secretaria de Vigilância em Saúde. Ministério da Saúde Brasil (2012) Especial Tuberculose 43 portal.saude.gov.br/portal/arquivos/…/bolepi_v43_especial_tb_correto.p…

[54] Piller RVB (2012) Epidemiologia da Tuberculose. Pulmão 121: 4–9 www.sopterj.com.br/revista/2012_21_1/02.pdf.

[55] Lago R F, Costa NR (2010) Policy dilemmas in providing antiretroviral treatment in Brazil. Ciênc Saúde Coletiva 15 supl 3: 3529–3540 http://dx.doi.org/10.1590/S1413-81232010000900028. 10.1590/s1413-81232010000900028 21120341

[56] Nunn AS, Fonseca EM, Bastos FI, Gruskin S, Salomon JA (2007) Evolution of antiretroviral drug costs in Brazil in the context of free and universal access to Aids treatment. PLoS Med 4: e305 http://www.plosmedicine.org/article/info:doi/10.1371/journal.pmed.0040305. 18001145 10.1371/journal.pmed.0040305 PMC2071936

[57] Budzik JM, Hosseini M, Mackinnon AC Jr, Taxy JB (2012) Disseminated Nocardia farcinica: literature review and fatal outcome in an immunocompetent patient. Surg Infect (Larchmt) 13: 163–170. 22612440 10.1089/sur.2011.012 PMC3375863

[58] Vuotto F, Faure K, Queyre V, Dessein R, Pasquet A, et al (2011) Vascular nosocomial *Nocardia farcinica* infection after arterial stenting in an immunocompetent patient. Can J Infect Dis Med Microbiol 22: e10–11. 22379487 10.1155/2011/216873 PMC3076156

[59] Hong SB, Han K, Son BR, Shin KS, Rim BC (2012) First case of *Nocardia nova* spinal abscess in an immunocompetent patient. Braz J Infect Dis 16: 196–199. 22552466 10.1016/s1413-8670(12)70306-9

[60] Lin CC, Lalitha P, Srinivasan M, Prajna NV, McLeod SD, et al (2012) Seasonal trends of microbial keratitis in South India. Cornea 31: 1123–1127. 22868629 10.1097/ICO.0b013e31825694d3 PMC4986607

[61] Mascarenhas J, Srinivasan M, Chen M, Rajaraman R, Ravindran M, et al (2012) Differentiation of etiologic agents of bacterial keratitis from presentation characteristics. Int Ophthalmol 32: 531–538. 22752605 10.1007/s10792-012-9601-x PMC3603562

[62] Nascimento EG, Carvalho MJ, de Freitas D, Campos M (1995) Nocardial keratitis following myopic keratomileusis. J Refract Surg 11: 210–211. 7553093

[63] Urbano AP, Urbano AP, Torigoe AMS, Urbano I, Kara-José N (2003) Spontaneus nocardial scleritis: case report. Arq Bras Oftalmol 66: 223–225.

[64] Hofling-Lima AL, Branco BC, Romano AC, Campos MQ, Moreira H, et al (2004) Corneal infections after implantation of intracorneal ring segments. Cornea 23: 547–9. 15256990 10.1097/01.ico.0000126434.95325.24

[65] Ramos-Esteban JC, Servat JJ, Silva RS, Ambrósio R Jr, Tauber S, et al (2007) Necrotizing nocardial scleritis after combined penetrating keratoplasty and phacoemulsification with intraocular lens implantation: a case report and review of the literature. Arq Bras Oftalmol 70: 355–359. 17589714 10.1590/s0004-27492007000200031

[66] Londero AT, Ramos CD, Matte SW (1986) Actinomycotic mycetomas in Rio Grande do Sul - report of 4 cases. Mem Inst Oswaldo Cruz 81: 73–7. 3796281 10.1590/s0074-02761986000100010

[67] Castro LG, Belda Júnior W, Salebian A, Cucé LC (1993) Mycetoma: a retrospective study of 41 cases seen in São Paulo, Brazil, from 1978 to 1989. Mycoses 36: 89–95. 8366881 10.1111/j.1439-0507.1993.tb00694.x

[68] Saraça GD, Towersey L, Hay RJ, Londero AT, Martins Ede C, et al (1993) Mycetoma by *Nocardia asteroides*: a 9 year follow-up. Rev Inst Med Trop Sao Paulo 35: 199–204. 8284606 10.1590/s0036-46651993000200013

[69] Lopes JO, Bassanesi MC, Alves SH, Salla A, Benevenga JP, et al (1994) Cutaneous *Nocardia asteroides* infection of nontraumatic origin. Rev Inst Med Trop Sao Paulo 36: 403–408. 7569606 10.1590/s0036-46651994000500003

[70] Lopes JO, Silva CB, Kmohan C, Oliveira LT, Dal Forno NL, et al (1995) Acute primary cutaneous *Nocardia asteroides* infection in a patient with systemic lupus erythematosus. Case report. Rev Inst Med Trop Sao Paulo 37: 547–50. 8731271 10.1590/s0036-46651995000600014

[71] Motta RL, Vilela RV, Lambertucci JR (2004) Actinomycetoma caused by *Nocardia brasiliensis* . Rev Soc Bras Med Trop 37: 287–288. 15330073 10.1590/s0037-86822004000300018

[72] Castro LG, Piquero-Casals J (2008) Clinical and mycologic findings and therapeutic outcome of 27 mycetoma patients from São Paulo, Brazil. Int J Dermatol 47: 160–163. 18211487 10.1111/j.1365-4632.2008.03447.x

[73] Dresch TFLR, Magalhães TC, Piñeiro-Maceira J, Akiti T, Ramos-e-Silva M (2010) Combined Therapy for Mycetoma: Medical and Surgical Dermatologic. Surgery 36: 952–954. 10.1111/j.1524-4725.2009.01418.x 20039916

[74] Magalhães GM, Oliveira SC, Soares AC, Machado-Pinto J, de Resende MA (2010) Mycetoma caused by *Nocardia caviae* in the first Brazilian patient. Int J Dermatol 49: 56–58. 20465613 10.1111/j.1365-4632.2009.04263.x

[75] Cordeiro F, Bruno C, Reis C (2011) Mycetoma. Am J Trop Med Hyg 85: 791. 22049027 10.4269/ajtmh.2011.10-0637 PMC3205619

[76] Brown-Elliott BA, Biehle J, Conville PS, Cohen S, Saubolle M, et al (2012) Sulfonamide resistance in isolates of *Nocardia* spp. from a US multicenter survey. J Clin Microbiol 50: 670–672. 22170936 10.1128/JCM.06243-11 PMC3295118

[77] Uhde KB, Pathak S, McCullum I Jr, Jannat-Khah DP, Shadomy SV, et al (2010) Antimicrobial-resistant nocardia isolates, United States, 1995–2004. Clin Infect Dis 51: 1445–1448. 21058914 10.1086/657399

[78] Tremblay J, Thibert L, Alarie I, Valiquette L, Pépin J (2011) Nocardiosis in Quebec, Canada, 1988–2008. Clin Microbiol Infect 17: 690–696. 20636427 10.1111/j.1469-0691.2010.03306.x

[79] No authors listed (2012) *Nocardia* resistant to trimethoprim-sulfamethoxazole? Maybe not. Clin Infect Dis 55: iii–iv. 22912969

[80] Brown BA, Lopes JO, Wilson RW, Costa JM, de Vargas AC, et al (1999) Disseminated *Nocardia pseudobrasiliensis* infection in a patient with AIDS in Brazil. Clin Infect Dis 28: 144–145. 10028089 10.1086/517180

[81] Severo CB, Oliveira FM, Cunha L, Cantarelli V, Severo LC (2005) Disseminated nocardiosis due to Nocardia farcinica: diagnosis by thyroid abscess culture. Rev Inst Med Trop S Paulo 47: 355–358. 16553327 10.1590/s0036-46652005000600009

[82] Bonnet F, Donay JL, Fieux F, Marie O, de Kerviler E, et al (2007) Postoperative nocardiosis caused by *Nocardia otitidiscaviarum*: pitfalls and delayed diagnosis. Ann Fr Anesth Reanim 26: 680–684. 17572044 10.1016/j.annfar.2007.03.032

[83] Petrillo VF, Severo LC, Londero AT, Porto NS (1978) Pulmonary nocardiosis report of the first two Brazilian cases. Mycopathologia 66: 17–20. 375092 10.1007/BF00429587

[84] Livramento JA, Machado LR (1989) Spina-França (1989) Anormalidades do líquido cefalorraqueano em 170 casos de AIDS. Arq Neuro-Psiquiatr 47: 326–331. 10.1590/s0004-282x1989000300013 2619610

[85] Coelho Filho JC (1990) Pulmonary cavities colonized by actinomycetes: report of six cases. Rev Inst Med Trop S Paulo 32: 63–66. 2259835 10.1590/s0036-46651990000100011

[86] Lopes JO, Alves SH, Benevenga JP, Salla A, Tatsch I (1993) *Nocardia asteroides* peritonitis during continuous ambulatory peritoneal dialysis. Rev Inst Med Trop Sao Paulo 35: 377–379. 8115800 10.1590/s0036-46651993000400013

[87] Aguiar PHP, Pahl FH, Uip DE, Vellutini EAS, Mutarelli EG, et al (1995) Abscesso cerebelar por Nocardia: relato de caso. Arq Neuro-Psiquiatr 53: 307–311. 10.1590/s0004-282x1995000200024 7487545

[88] Machado CM, Macedo MC, Castelli JB, Ostronoff M, Silva AC, et al (1997) Clinical features and successful recovery from disseminated nocardiosis after BMT. Bone Marrow Transplant 19: 81–82. 9012936 10.1038/sj.bmt.1700616

[89] Camargo LACR, Silva Junior EF, Lapchik MS, Di Loreto C (1997) Pulmonary nocardiosis: presentation of a clinical case with poor evolution. J Pneumol 23: 211–214.

[90] Barata CH, Oliveira DA, Colombo AL, Pereira CA (2000) Brain abscess caused by *Nocardia* sp in immunosuppressed patient. Rev Soc Bras Med Trop 33: 609–612. 11175594 10.1590/s0037-86822000000600015

[91] Silva ACG, Martins EML, Marchiori E, Torres Neto G (2002) Nocardiose pulmonar em paciente com síndrome da imunodeficiência adquirida: relato de caso. Radiol Bras 35: 235–238.

[92] Faucz RA, Quadros MS, Andrade CA, Troncoso FT, Ribeiro Filho NF, et al (2006) Triple pulmonary infection in a severely immunocompromised AIDS patient: a case report. Radiol Bras 39: 79–82.

[93] Baldi BG, Santana AN, Takagaki TY (2006) Pulmonary and cutaneous nocardiosis in a patient treated with corticosteroids. J Bras Pneumol 32: 592–595. 17435912 10.1590/s1806-37132006000600019

[94] Brasileiro RMF, Pinho ACCA, Medeiros CS, Ferri F, Schiavon LL, et al (2007) Pulmonary nocardiosis in a patient who was a chronic corticosteroid user. Rev Soc Bras Med Trop 40: 585–587. 17992418 10.1590/s0037-86822007000500018

[95] Aidê MA, Lourenço SS, Marchiori E, Zanetti G, Mondino PJJ (2008) Nocardiose pulmonar em portador de doença pulmonar obstrutiva crônica e bronquiectasias. J Bras Pneumol 34: 985–988. 19099108 10.1590/s1806-37132008001100016

[96] Moraes PRS, Chimara E, Telles MAS, Ueki SYM, Cunha EAT, et al (2008) Identification of non-tuberculous mycobacteria from the Central Public Health Laboratory from Mato Grosso do Sul and analysis of clinical relevance. Braz J Microbiol 39: 268–272. 24031214 10.1590/S1517-838220080002000013 PMC3768383

[97] Sarcinelli-Luz B, Marchiori E, Zanetti G, Mano CM, Abdalla F, et al (2009) Pulmonary nocardiosis in the acquired immunodeficiency syndrome, computed tomographic findings: a case report. Cases J 15: 6642. 10.1186/1757-1626-2-6642 PMC2740044 19829838

[98] Rêgo RSM, Silveira NSS, Lima KM, Melo FM (2009) Disseminated nocardiosis in leucemic patient. Rev Bras Anal Clin 41: 51–53.

[99] Castelli JB, Siciliano RF, Abdala E, Aiello VD (2011) Infectious endocarditis caused by *Nocardia* sp.: histological morphology as a guide for the specific diagnosis. Braz J Infect Dis 15: 384–386. 21861012

